# EFOMP: the European roof for medical physics

**DOI:** 10.2349/biij.3.3.e32

**Published:** 2007-07-01

**Authors:** W Schlegel

**Affiliations:** Department of Medical Physics in Radio-Oncology, Deutsches Krebsforschungszentrum (dkfz), Heidelberg, Germany

**Keywords:** EFOMP, medical physics, education and training, professional affairs

## HISTORY

Medical Physics is a field of applied physics and as such a comparatively young discipline. The roots of Medical Physics go back to the application of ionising radiation in diagnostics and therapy in the beginning of the last century. Between the years 1950 and 1970, the increasing importance and specialisation of Medical Physics led to the foundation of Medical Physics societies in most European countries. Currently, nearly all European states have Medical Physics societies, with more than 6,000 personal memberships.

**Figure 1:**
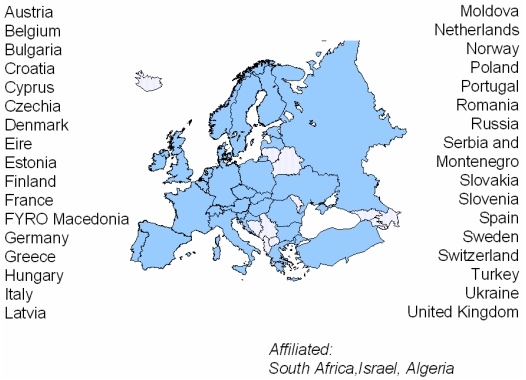
National member organisations of EFOMP.

Due to an initiative of the English hospital physicists association (HPA), there was a suggestion in 1979 to form a European Roof organisation that included all national societies. In May 1980, this proposal led to the foundation of the “European Federation of Organisations in Medical Physics” (EFOMP) as an association of the European national societies in Medical Physics, which, in the EFOMP nomenclature are called “National Member Organisations (NMOs)”. Starting with 14 foundation member states, EFOMP today has 35 NMOs, which are not only restricted to the area of the European Community (EC), but also includes non-EC-member states such as Switzerland, and in the meanwhile also includes most East-European countries, and associated countries like Israel, Algeria and South Africa (Figure 1).

The reason for the foundation of a European roof organisation was due to the fact that Medical Physics was practiced in the different states in quite a different way. In European countries, there was a large variation in education and training that was very differing or even missing regulations, and a very underdeveloped scientific collaboration between the countries.

## STRUCTURE

**Figure 2:**
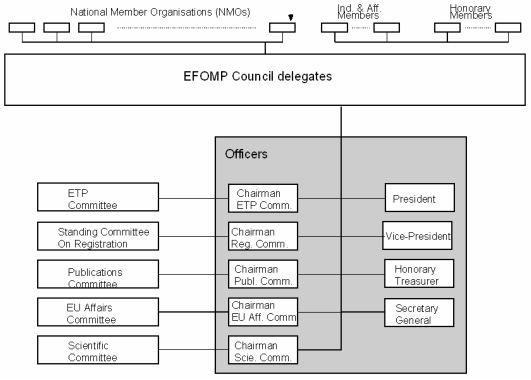
Structure of EFOMP.

The structure of EFOMP is shown in Figure 2. The principal group of EFOMP members are the National Medical Physics Organisations. In addition, EFOMP has Honorary Members – individual medical physicists who have made a significant contribution to the field, and industrial members, which are commercial companies wishing to support the Federation.

The ruling body of EFOMP is the Council, which meets annually. The Council is made up of representatives of NMOs (two for each NMO), all of whom have votes. The number of votes (from 1 to 3) cast by a given NMO is determined by its size.

The Council elects a Board of Officers, responsible for the running of the Federation in accordance with the Council’s wishes. The Officers comprise the President, Vice-President, Treasurer, Secretary-General and the Chairmen of the Federation’s Committees.

## MISSION

Due to the EFOMP statutes, the mission of the Federation is, first of all, to harmonise and advance Medical Physics at an utmost level in its professional, clinical and scientific expression throughout Europe; second, to strengthen and make the activities of the NMOs more effective by bringing about and maintaining a systematic exchange of professional and scientific information, and by the formulation of common policies, and last but not least by promoting education and training programmes.

## TASKS

**Figure 3:**
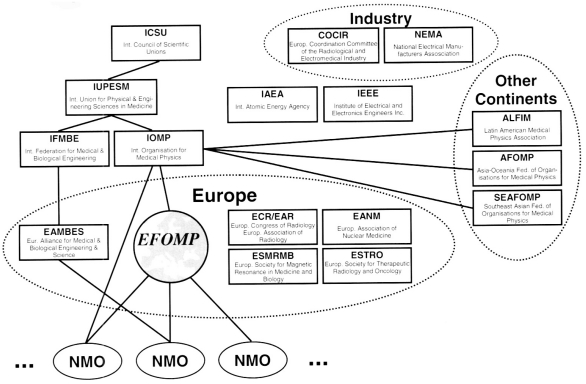
Organisations around EFOMP.

According to the mission, tasks of EFOMP are:

1. To promote science in the Medical Physics area, e.g. by cooperating and interacting with other organisations (like the ESR, EANM, ESTRO, ESMRMB and others, see Figure 3), by organising EFOMP congresses and by the support of meetings and courses. A new initiative in this context is the EFOMP Journal “Physica Medica/ European Journal of Medical Physics (EJMP)”. Publishing of the EJMP is currently being started by Elsevier. The scope of the journal is described in Table 1.
2. To promote and harmonise European education and training in Medical Physics. EFOMP has been publishing guidelines and policy statements [5, 6, 7, 10], set up working groups [3, 9], and together with the European Scientific Institute (ESI) running the European School of Medical Physics (ESMP) in Archamps/France. ESMP has been continuously running for 10 years now with 5-week courses (see Table 2).
3. Last but not least, EFOMP is promoting the Medical Physics profession and best practice of Medical Physics in Europe, mainly by publishing guidelines and policy statements by EFOMP, and by registering national education and training programmes [1, 2, 4, 8].

**Table 1:** Scope of the official EFOMP journal Physica Medica (see: http://www.elsevier.com/wps/find/journaldescription.cws_home/712167/description#description).

**“Physica Medica / European Journal of Medical Physics”** Medical Imaging Radiation Therapy Radiation Protection Measuring Systems and Signal Processing Education and training in Medical Physics

**Table 2:** Themes and topics of the 5-week courses of the European School for Medical Physics held yearly in Archamps/France (see: http://lemoigne.web.cern.ch/lemoigne/esiweb/esmpn1.htm).

**Week 1** Medical Imaging-1-: Principles, Ultrasound & Magnetic Resonance	Principles of magnetic resonance Basis & examples of Biometry. Magnetic resonance imaging & magnetic resonance spectroscopy. Clinical & anatomy MRI Advanced MRI & applications Basics Physics & engineering Principles of ultrasound. Doppler Methods. Clinical applications and biological aspects of ultrasound
**Week 2** Medical Imaging-2-: Medical imaging with Ionising Radiation	Image formation (concepts and math. theory) and image quality (theory, evaluation). Advances in radiation detectors and Electronic signal treatment Digital imaging and X- Computer Tomography, Mammography. Image processing & storage. Principle of SPECT & PET. Production & use of radioisotopes. Advance PET & applications; Quality control & performance assessments.
**Week 3** Medical Computing	Simulation/modelling of physics systems, courses and exercises. Simulation/modelling of live systems: courses and exercises (GEANT4, EGS4, EGSnrc...) Simulation and Pharmacokinetics Imaging; processing; use of images and data Networks; data communications Practical exercises on Computer
**Week 4** Physics of Modern Radiotherapy	Fundamentals of radiobiology. Radiotherapy strategies. Accuracy requirements. Imaging in treatment planning. Treatment planning: courses and exercises. Conformal treatment techniques. Hadrontherapy. Clinical application of 3D conformal therapy.
**Week 5** Brachytherapy	Brachytherapy sources; calibration and quality assurance Dosimetry for photon sources & beta sources; dose calculation models. Treatment units for HDR/LDR/PDR; reporting in Brachytherapy. BT for benign disorders; BT for malignant disorders. Radiation protection and quality assurance. Practical exercises : treatment planning for some medical cases

## COMMITTEES

The operative work of EFOMP is performed via its committees.

EFOMP’s officers chair the five Committees consisting of core and corresponding members. The functions of each Committee are as follows:

### The Education, Training and Professional (ETP) Committee

The ETP Committee deals with issues of physicist education and training, and with many of the matters surrounding the responsibilities and roles of medical physicists. The successful EFOMP Summer Schools are organised largely by ETP-Committee working groups.

### The Scientific Committee

The Scientific Committee is responsible for EFOMP’s activities in furthering the science of medical physics. The scientific committee is the body within EFOMP which organises conferences and congresses (see Table 3), nominates members to program and organising committees and which liaises with external scientific organisations such as the European Congress of Radiology.

**Table 3:** EFOMP congresses between 1985 and 2009.

**City/ Country**	**Year**
Innsbruck/Austria	1987
Oxford/ United Kingdom	1990
Tenerife/ Spain	1993
Wuerzburg/Germany	1995
Trieste/Italy	1996
Patras/ Greece	1999
Belfast/ Ireland	2001
Eindhoven/Netherlands	2003
Nuremberg/Germany	2005
Pisa/Italy	2007
Munich/Germany	2009

### The Standing Committee on Registration Matters

The Standing Committee on Registration Matters implements and develops EFOMP’s registration scheme of national registration schemes.

### The Committee on European Union Affairs

The Committee on European Union Affairs recognises the growing importance of EU policies even to physicists in non-EU countries.

### The Communications and Publications Committee

The Communications and Publications Committee is responsible for disseminating information, both to EFOMP members and to the wider public. The chairman is also the webmaster of the EFOMP website.

## FUTURE GOALS OF EFOMP

There are three important fields of activities within EFOMP: education and training, professional affairs and science.

### Education and training

As far as education and training is concerned, the goal is to promote and harmonise European education and training in Medical Physics. The current situation in education and training in Europe was investigated by the EFOMP-ETP committee during the last 3 years, and it turned out that regular education and training programs are missing in most European countries and further harmonisation is urgently required [10].

A recent activity within an ESTRO/EFOMP working group (2002-2003) was to establish guidelines for education and training of medical physicists in radiation oncology [9]. Another group is working on guidelines for education and training of medical physicists in radiology since 2006.

A future activity of EFOMP is to establish, in cooperation with the NMOs, a European network of Pro Medical Physics Training Schools (ENMPS).

### Professional affairs of Medical Physics

Promoting the Medical Physics profession and best practice of Medical Physics in Europe is the second-most important aim of EFOMP. The encountered problems are Medical Physics is currently not listed on the list of professions of the International Labour Organisation (ILO), and it is also not registered as an official profession in most European countries. As a consequence, mobility of Medical Physicists between countries is difficult to perform. In the frame of a declaration, which was worked out in the last council meeting in Malaga (the so called Malaga declaration, see www.efomp.org), EFOMP is trying to introduce Medical Physics as a health profession on the ILO list. The Malaga declaration also includes a statement that the responsibility for radiation protection in hospitals has to be taken over by Medical Physicists.

### Science in Medical Physics

The third field of concern is Medical Physics as a science in Europe. There are two major problems, first, there are only very few independent departments or Chairs for Medical Physics at European Universities or research centres, and second, the scientific activities in Medical Physics are distributed to many different medical societies (ESTRO, EANM, ESMRBM, ESR, etc.). This situation makes interdisciplinary collaborations difficult. EFOMP’s goal is to strengthen Medical Physics science in Europe, and there are different approaches currently being discussed, e.g. changing the legal status of EFOMP in order to enable participation in EU-funded projects, establishing scientific communication and publication platforms, and inaugurating a strong scientific “European Congress on Medical Physics”. Such a first, “European Congress in Medical Physics”, solely organised by EFOMP, is now being organised to take place in Pisa, Italy, in September 2007.
